# COVID-19 Pandemic Impact on the Birth Weight of Children Born in a Brazilian Metropolis

**DOI:** 10.3390/ijerph21121702

**Published:** 2024-12-20

**Authors:** Beatriz Cardoso Armani, Rafaela Cristina Vieira e Souza, Fernanda Penido Matozinhos, Luana Caroline dos Santos

**Affiliations:** 1 Nutrition Department, Nursing School, Federal University of Minas Gerais, Belo Horizonte, MG 30130-100, Brazil; rafasouzacec@gmail.com (R.C.V.e.S.); luanacstos@gmail.com (L.C.d.S.); 2 Maternal and Child Nursing and Public Health Department, Nursing School, Federal University of Minas Gerais, Belo Horizonte, MG 30130-100, Brazil; nandapenido@hotmail.com

**Keywords:** birth weight, pregnancy, COVID-19, food intake, exercising

## Abstract

Objective: To assess the birth weight of newborns whose mothers gave birth during the COVID-19 pandemic. Methods: A cross-sectional study based on data collected from medical records and through postnatal interviews to assess maternal and neonatal health outcomes (n = 470) during the pandemic. All participants were assisted in three Brazilian public hospitals in 2020. Multinomial logistic regression was performed to assess factors associated with birth weight. Results: Low and insufficient birth weight reached 9.8% and 25.7% prevalence, respectively. COVID-19 symptoms were reported by 8% of participants. Low birth weight was more often observed in premature children (OR: 70.9; 95% CI: 16.4–305.8) delivered by cesarean sections (OR: 7.70; 95% CI: 2.33–25.4). Insufficient weight was more frequent in premature children (OR: 5.59; 95% CI: 1.53–20.4) and children whose mothers did not exercise during pregnancy (OR: 2.85; 95% CI: 1.38–5.89). Women presenting higher gestational weight gain had a lower chance of delivering babies with insufficient weight (OR: 0.94; 95% CI: 0.90–0.99). Conclusions: Inadequate birth weight was associated with prematurity, delivery type, lower gestational weight gain, and maternal physical inactivity during the pandemic. According to the results, it is necessary to have adequate prenatal care and promote a healthy lifestyle during pregnancy.

## 1. Introduction

COVID-19 is the disease caused by SARS-CoV-2 coronavirus, and human cases of it were reported for the first time in late 2019 in Wuhan City, China [1]. In March 2020, this disease was declared a pandemic by the World Health Organization (WHO). Brazil was one of the most affected countries and recorded 36,331,281 cases by late 2022 [1,2]. Some groups, such as pregnant women, who had a higher risk of complications, were considered particularly vulnerable to this infection [3].

Because of physiological and anatomical changes like immunological adaptation conditions, higher oxygen consumption, and reduced lung capacity, pregnant women are group that requires closer attention during infectious-disease outbreaks [4,5]. Zambrano et al. have shown that this population was more often admitted to intensive care units during the COVID-19 pandemic and these women were subjected to more invasive ventilation, in addition to facing an increased risk of death. According to a study conducted in England, SARS-Cov-2 infection was associated with higher preeclampsia rates and cesarean section prevalence than pregnant women without it [6].

Normal fetal development could also be influenced by SARS-CoV-2 infection since it could lead to outcomes like preterm birth and low birth weight [7,8]; furthermore, fetal COVID-19 complications include intrauterine growth limitations [9]. Birth weight is known as an important neonatal health indicator and low birth weight is a risk factor for perinatal morbidity and lifelong health complications [10]. The pandemic highlighted that COVID-19 consequences exceeded the morbidity caused by this, and had a significant socioeconomic impact on healthcare factors such as income, food security, social isolation, fear of virus contamination and number of prenatal visits, with a notable influence on birth weight [11,12,13].

According to a report by the Food and Agriculture Organization of the United Nations (FAO), nearly 12% of the global population faced severe food insecurity in 2020 [14]. This increase is attributed to various factors, including disruptions in food supply chains that elevated prices, social isolation that restricted physical access to food, and income reductions resulting from the decline in economic activities [15,16]. Studies indicate that food insecurity during pregnancy can result in either insufficient or excessive gestational weight gain, both of which are detrimental to maternal and fetal health. Nutrient deprivation is frequently associated with low gestational weight gain [17]. On the other hand, food insecurity can also lead to increased consumption of ultra-processed and energy-dense foods, resulting in excessive gestational weight gain [18]. Both extremes pose significant risks and are associated with adverse outcomes such as preterm birth, low birth weight, and macrosomia [16,18].

Moreover, maternal mental health became particularly vulnerable during the pandemic due to social isolation and uncertainty regarding neonatal outcomes. In one of the first systematic reviews published on the mental health of pregnant and postpartum women during the pandemic, an increase in the prevalence rates of anxiety, depression, and insomnia among pregnant women was observed compared to the pre-pandemic period [19]. Other studies have shown that symptoms of depression and anxiety can increase the risk of adverse pregnancy outcomes, such as low birth weight [20,21].

According to a systematic review, 15% of babies born from pregnant women who tested positive for COVID-19 recorded low birth weight [22]. However, a prospective cohort did not identify differences in birth weight when this outcome was compared based on the presence of Sars-Cov-2 infection [23]. Given the scarcity of studies on this topic and the hypothesis that COVID-19-related disruptions can affect birth weight, the aim of the present study was to assess the pandemic’s impact on the birth weight of babies born at that time.

## 2. Materials and Methods

This was a cross-sectional study based on data collected during the first two stages of a cohort study entitled “Childbirth and breastfeeding applied to children of mothers infected with SARS-CoV-2”. The first stage aimed to collect data from medical records of women who gave birth in three public hospitals in Belo Horizonte City, Brazil, in the first three months of high COVID-19 incidence (May, June, and July) in 2020 [24]. Gestational and clinical data, such as birth weight, gestational age at birth, number of prenatal consultations, parity, and newborn sex, were collected at this stage. Women were addressed by phone, between the 6th and 11th months of pregnancy, and 29 days after delivery. At the second stage, we collected additional sociodemographic information: income and marital status, record of exercising, nutritional monitoring during pregnancy, alcohol consumption, smoking, and food intake.

**Figure 1:**
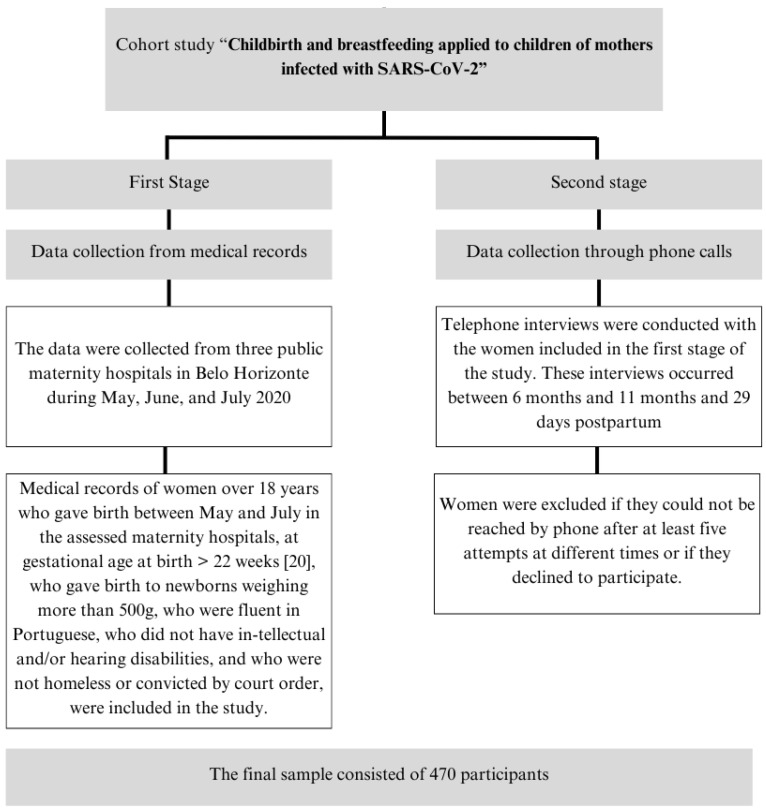
Design of the present study.

Sample size calculation was based on an expected rate (31%) of newborns presenting low birth weight and insufficient weight in Brazil, and was substantiated by data from a pre-pandemic period. Wall tests adopted a 95% confidence interval (CI) and a 5% error margin. The initial estimate pointed towards 330 participants, and the final sample comprised 470 participants [25] (Figure 1).

### 2.1. Inclusion and Exclusion Criteria

Medical records of women over 18 years who gave birth between May and July in the assessed maternity hospitals, at gestational age at birth > 22 weeks [26], who gave birth to newborns weighing more than 500 g, who were fluent in Portuguese, who did not have intellectual and/or hearing disabilities, and who were not homeless or convicted by court order were included in the study.

### 2.2. Data Collection

#### 2.2.1. Sociodemographic Data

The following sociodemographic data were collected through a questionnaire adapted from a study based on similar public targets [27]: age (in years), income (no income, up to 1 minimum wage, 1 to 3 minimum wages, 3 to 5 minimum wages or more than 5 minimum wages), schooling (illiterate, primary school, elementary school, high school or higher education) [28], marital status (single, married, in a stable relationship for more than six months, widowed or divorced), paid work before the pandemic (yes or no), and self-declared skin color (white, black, brown, Asian descendant or Indigenous) [29].

#### 2.2.2. Pregnancy and Delivery Data

Information on pregnancy and delivery included birth weight, gestational weight-gain (GWG) in kilograms, delivery route (vaginal or cesarean section), gestational age at birth expressed in weeks and days (preterm or full-term), parity (1 child, and 2 or more children), total number of prenatal consultations (less than 6 consultations, and 6 or more consultations [30]), whether there was follow-up with dieticians (yes or no) and if there was any diabetes mellitus diagnosis during pregnancy (yes or no). Birth weight was classified as low (<2500 g), insufficient (between 2500 g and 3000 g), and adequate (>3000 g) [31,32,33].

#### 2.2.3. Life Style

Smoking (yes or no), alcohol consumption (yes or no), and exercising (yes or no) data during pregnancy were also collected.

### 2.3. Pandemic Context

#### 2.3.1. COVID-19 Symptoms

We also analyzed data about COVID-19 symptoms during pregnancy (yes or no), such as fever, cough, and sore throat, due to the low number of tests in Brazil, in 2020, according to the “Clinical Management Protocol for Coronavirus in Primary Health Care” [34].

#### 2.3.2. Food Consumption

A questionnaire about food intake frequency markers during the gestational period, based on national research [35,36,37] and studies focused on food intake during the pandemic in Brazil, was adopted [38]. Nine food type categories were included: ready-to-eat food (prepared outside the home); beans, peas, chickpeas or legumes; fresh fruit (fresh fruit juices were not considered); vegetables and/or legumes (potatoes, cassava and yam were not taken into account); processed meat (burgers, ham, salami, sausage, hot dogs); sweetened beverages (sugary fruit juice, soda, canned juice, powder juice, caned coconut water, guaraná/blackcurrant syrup); instant noodles, packaged snacks or crackers; stuffed cookies and sweets (candies/sweets, chewing gum, jelly, brigadeiro, sweet pies); and, finally, beef, pork, chicken or fish. Frequencies applied for reporting purposes included “never”, “1 to 2 times a month”, “2 times a week”, “3 to 4 times a week”, “5 to 6 times a week”, and “every day”.

Food intake was classified into four categories [39]: “fresh or minimally processed food”, “culinary ingredients” (salt, sugar, oils and fats used for cooking), “processed food” (fresh or minimally processed food by using salt or sugar, or other culinary-use substances) and “ultra-processed food” (industrial formulations or food mainly prepared with substances extracted from food added with several additives to provide attractive sensory properties to these products).

The consumption of fresh and minimally processed food for five or more days a week was considered regular, while consuming ultra-processed food for two or more days a week was considered inadequate [40,41].

### 2.4. Statistical Analysis

A database was created based on double data entry in Microsoft Office Excel 2016, and consistency analysis was conducted. The Kolmogorov–Smirnov normality test was used to assess the distribution of numerical variables expressed as median and interquartile range (IQR). Categorical variables were described through absolute and relative frequencies.

Spearman’s correlation test was performed to compare independent variables to birth weight taken as a continuous variable. Kruskal–Wallis and Mann–Whitney tests allowed the comparison of the outcome based on qualitative variable categories (income, schooling, marital status, exercising, consultation with a dietician, COVID-19 symptoms and food intake). A chi-square test with Bonferroni correction was run to compare the categorical variables, whenever necessary. Multinomial logistic regression was performed to assess factors associated with birth weight. All variables recording *p* < 0.20 in the bivariate analysis were included in the regression model based on backward elimination. This procedure regards the sequential elimination of the least significant variables in the model, starting with the one accounting for the highest *p*-value. In addition, multivariate analyses were adjusted to maternal age, schooling, smoking, alcohol use, and gestational diabetes mellitus. These adjustments were essential to control the impact of the assessed variables on the primary outcome to reach a more accurate analysis of associations between variables of interest and to minimize potential biases. Well-documented variables related to the primary outcome in the literature were selected to be adjusted. The analyses were performed in Statistical Package for Social Sciences (SPSS), version 21.0, and in Stata software, version 14.0, at a 5% significance level.

### 2.5. Aesthetical Aspects

The study “Childbirth and breastfeeding in children of mothers infected with SARS-CoV-2”, which encompasses the current study, was approved by the Research Ethics Committee of Federal University of Minas Gerais (Opinion n. CAAE: 32378920.6.1001.5149). All postpartum women and managers of each assessed maternity hospitals signed the Free and Informed Consent Form, according to the ethical guidelines described in Resolution n. 466/2012 of the National Health Council.

## 3. Results

### 3.1. General Sample Features

In total, 470 women participated in the study. They recorded a median age of 27 years (IQR 9). Most of them had at most a high school degree (85.3%), brown skin (57.2%), and a partner (62.6%). Approximately 8% of pregnant women reported COVID-19 symptoms before delivery and 29.6% of them had a cesarean section.

### 3.2. Pregnancy and Childbirth

In total, 9.8% of newborns recorded low birth weight and 25.7% of them were born with insufficient weight. These results are similar to those found in Brazil in 2019, during the pre-pandemic period. In that year, 8.5% of newborns had low birth weight, and 22.6% were born with insufficient weight [25]. A higher frequency of low birth weight was observed in babies born by cesarean section (15.8% vs. 7.2%) compared to those with adequate weight (52.5% vs. 69.5%). Additionally, the prevalence of low birth weight was significantly higher in preterm babies (62.8% vs. 4.3%) compared to those with adequate weight (14% vs. 69.5%). Insufficient birth weight was more prevalent among women who did not exercise during pregnancy (28.8% vs. 20.8%) compared to those with adequate-weight babies (59.9% vs. 71.9%). There was no association between birth weight and other tested variables (Table 1).

Gestational weight gain, gestational age, and birth weight medians were 10 kg (IQR 8), 39 weeks (IQR 2), and 3170 g (IQR 660), respectively. A higher weight median was identified in babies of pregnant women who had vaginal delivery [3205 g (IQR 632) vs. 3040 g (IQR 778); *p* = 0.006] and in babies born at term [3210 g (IQR 617) vs. 2390 g (IQR 970); *p* < 0.001]. There was a correlation between birth weight and gestational age (r = 0.421; *p* < 0.001), and gestational weight gain (r = 0.171; *p* = 0.001), when the main outcome was taken as a continuous variable.

### 3.3. Food Intake

The most common foods consumed during pregnancy during the pandemic were beans (88.2%), vegetables and/or legumes (74.5%), and meat (77.0%). In total, of the consumed ultra-processed foods, the sweetened, prepared food, and food shops and restaurants categories accounted for 66.2% and 21.2%, respectively. Based on the present results, and after Bonferroni correction, there was no association between birth weight categories and food intake (Table 2).

### 3.4. Multivariate Analysis

Higher chance of low birth weight in babies born by cesarean section was observed through multivariate analysis (OR: 7.70; 95% CI: 2.33–25.4), as well as in preterm babies (OR: 70.9; 95% CI: 16.4–305.8) after adjustments for maternal age, schooling, smoking, alcohol consumption, and diabetes mellitus during pregnancy. Insufficient weight was more often observed in preterm children (OR: 5.59; 95% CI: 1.53–20.4) and in children whose mothers did not exercise during pregnancy (OR: 2.85; 95% CI: 1.38–5.89). There was a lower chance of inadequate weight in women with higher gestational weight gain (OR: 0.94; 95% CI: 0.90–0.99) after these adjustments (Table 3).

## 4. Discussion

According to the results of the present study, there was a high prevalence (35.5%) of inadequate birth weight during the pandemic. Low birth weight was associated with prematurity and cesarean section. Insufficient birth weight was associated with premature birth and lack of exercise during pregnancy. The chance of inadequate birth weight was lower in women presenting high gestational weight gain.

A similarly low birth weight rate (10.2%) was observed in babies of Brazilian women, regardless of COVID-19 positive or negative test results [42]. In 2019, in the pre-pandemic period, Brazil recorded 8.5% of births weighing less than 2500 g and 22.6% weighing between 2500 g and 3000 g; these findings are consistent with those observed in our study [25]. Inadequate birth weight is an important public health indicator and this condition is closely associated with infant mortality, mainly with maternal health and nutrition [42,43,44]. The literature showed divergences between perinatal outcomes recorded during the pandemic. Some of these studies highlighted higher low-birth-weight frequency [7,8,23] in women infected with SARS-CoV-2. These findings likely result from lifestyle changes observed at that time, such as diets composed of a lower intake of fresh food and less exercising, difficulty accessing prenatal care, and social vulnerability [45,46,47,48]. A study carried out in the United States showed that changes in prenatal care during the COVID-19 pandemic led to significant emotional impacts, mostly fear and anxiety related to prenatal care and childbirth [49]. Higher anxiety levels were notably linked to lower birth weight in newborns [50]. Other studies did not identify significant differences in outcomes, as observed in our work [51]. These findings point towards the multifactorial nature of birth weight, since it encompasses prenatal care and maternal health, as well as socioeconomic and lifestyle issues like diet and exercising.

According to the current analyses, inadequate birth weight was favored by prematurity, among other factors, and it can be explained by both pregnancy shortening and shorter fetal growth time [52,53]. In total, 9.6% of births in the present sample were premature, and this rate is similar to that found in a retrospective study on births in Brazil between 2012 and 2019 [54]. The 2020 global prematurity rate was very similar to the aforementioned one (9.9%) [55]. A recent systematic review did not show an association between preterm birth and SARS-Cov-2 infection [45], and data in the current study were also similar to those recorded during the pre-pandemic period.

In addition to prematurity, cesarean section was also associated with low birth weight. Cesarean sections can benefit the health of mother and baby whenever it is well recommended. An analysis of publicly funded hospitals in Southeastern Brazil recorded a cesarean section rate similar to the one observed herein (24.6%) [56]. A survey carried out in China [57] analyzed the delivery type and neonatal outcomes, and showed that higher cesarean section rates were linked to low-birth-weight newborns. The association between cesarean sections and low birth weight can be explained by iatrogenic practices related to elective cesarean sections often performed before labor onset [58]. Lower birth weight may be associated with cesarean delivery due to preexisting pregnancy complications that lead to a surgical indication, such as placental abruption, umbilical cord prolapse, placenta previa, chronic fetal distress, uncontrolled hypertensive disorders, and other obstetric emergencies [59,60]. Studies have shown an expected increase in the number of cesarean sections during the COVID-19 pandemic [61], including in Brazil, although this number has been growing since 2017. Increased maternal requests due to uncertainties related to this disease, lower quality of prenatal care due to social isolation, restricted presence of a partner during childbirth hospitalization, and the fact that pregnant women arrived at maternity hospitals with advanced obstetric complications are among the reasons for this trend [62,63].

However, based on the current results, the cesarean section rate was similar to that recorded in the pre-epidemic period, and the same result was recorded in a British hospital [63]. This finding is likely justified by the fact that hospitals, in Brazil, where data were collected, are references in maternal and child care. Furthermore, 80% of the present sample attended at least six prenatal consultations, and it helped reduce complications and the number of elective cesarean sections. We recommend that future studies compare birth weight in elective cesarean sections versus emergency cesarean sections, considering that the factors leading to the indication for surgical delivery may act as a potential confounding factor in the association between low birth weight and cesarean delivery.

Lack of exercise during pregnancy was associated with insufficient birth weight, and this finding corroborated current recommendations for exercising during pregnancy [64]. A review highlighted that regular exercise is protective against birth weight extremes due to improved placental blood flow. Furthermore, it helps to control pregnant women’s body weight [65]. Approximately 22% of American pregnant women reduced their exercising time due to social isolation [66]. On the other hand, 20.6% of women in a Brazilian study reported having engaged in exercise at some point during pregnancy during the pre-pandemic period. The current sample reported an even higher rate, and this finding can be explained by the growing concern with health during pregnancy, mainly during the pandemic. It is important to point out that exercising can be influenced by socioeconomic factors. A Brazilian study highlighted that pregnant women with lower income and educational levels undertook less exercise during pregnancy due to limited access to resources, limited information about the benefits of exercising, or structural barriers, such as lack of both time and social support [67].

Studies have been shown about the association between gestational weight gain and fetal growth [68,69]. According to data in the present research, women who presented higher weight gain during pregnancy were less likely to give birth to a newborn weighing less than 2500 g. Inadequate weight gain is associated with low birth weight and prematurity, whereas excessive weight gain increases the risk of macrosomia [69,70]. Although data in the present study did not allow the classification of gestational weight gain as adequate or inadequate due to a lack of pre-gestational anthropometric information, they reinforced the ability of this potentially modifiable factor in determining birth weight.

The sample in the current study recorded a small number of performed tests and made it essential to analyze the features of SARS-CoV-2 infection symptoms. No differences were found in the birth weight of children whose mothers presented COVID-19 symptoms during pregnancy. It is known that asymptomatic SARS-CoV-2 transmission is also real, but it is more difficult to identify at times of health crisis [71]. However, studies have shown that symptoms, such as cough, fever, and smell and taste loss, are potential COVID-19 predictors [72].

It is worth noting that birth weight assessment happened at early pandemic stages, but, due to its long duration, results could have been different in other studies. It is assumed that the socioeconomic impacts of this context will be observed for years from now, and makes further longitudinal studies necessary to assess the pandemic’s repercussions on maternal and child health.

The limitations of the present study include the fact that the sample, despite comprising three large public maternity hospitals, was not representative of the pregnant women population in the assessed city. Furthermore, inclusion criteria “being fluent in Portuguese” and “literate” may have limited the sample’s scope. The small number of pregnant women who reported COVID-19 symptoms can also be considered a limitation since it might have restricted the ability to associate this variable with birth weight. It is noted that the interviewed women may not have accurately recalled information about the gestational period, as the telephone interviews took place at least six months after delivery. Over time, pregnancy-related events are more likely to be underreported, and the way questions are formulated can influence responses, especially regarding subjective variables, such as dietary habits and physical activity during pregnancy. A Brazilian study compared subjective methods, such as questionnaires, and objective methods, such as pedometers, for measuring physical activity during pregnancy and found that questionnaires tend to overestimate this practice [73]. Therefore, to mitigate this bias, the use of structured questionnaires is recommended, as they can improve data reliability, along with the combination of different sources of information, such as medical records or objective methods, whenever possible. This recall bias may have influenced our results, such as the fact that no dietary intake variable was eligible for multivariate analyses. Finally, it should be noted that the results presented only showed an association between the analyzed variables and did not establish causal associations due to the cross-sectional design. To investigate these causal relationships more thoroughly, longitudinal studies are recommended to deeply investigate these causal associations, as they allow an analysis of changes taking place over time and a more accurate understanding of factors influencing the observed outcomes. The current study included a population that was particularly vulnerable to the pandemic, besides being the first to investigate the birth weight of children born during one of the most critical periods of the COVID-19 pandemic in the assessed metropolis.

## 5. Conclusions

Inadequate birth weight prevalence was high in the first months of the pandemic in Brazil, although the recorded numbers were similar to the pre-pandemic ones. Birth weight inadequacies were associated with prematurity, delivery route, lower gestational weight gain, and maternal lack of exercise during pregnancy. These findings highlight the multifaceted factors influencing birth weight, regardless of the health conditions, besides emphasizing the need for interventions to ensure adequate prenatal care and nutritional support. Therefore, public policies are needed to expand access to prenatal care, especially in more vulnerable populations. It is essential to strengthen strategies to ensure access to adequate nutrition and nutritional counseling. The public health system, which includes nutrition professionals, can expand the availability of services such as group discussions on healthy eating. Additionally, physical education professionals present in healthcare facilities can develop specific strategies for this group, such as informational booklets on the importance of physical activity during pregnancy. These actions strengthen health care at a crucial time, helping to prevent adverse outcomes such as inadequate birth weight.

Future research could broaden the sample to include different regions and socioeconomic contexts in the analysis, and take into consideration data previously collected during the pandemic, as well as incorporate gestational information, such as pre-gestational body mass index. Furthermore, it is essential to analyze the pandemic’s long-term impact on maternal and child health based on gestational conditions experienced at that time.

## Figures and Tables

**Table 1:** Socioeconomic and gestational features of births registered in the assessed maternity hospitals between May and July, 2020.

Variables	Total Sample (n%)	Adequate Weight (n%)	Insufficient Weight (n%)	Low Weight (n%)	*p*-Value *
Schooling	470	303 (64.5)	121 (25.7)	46 (9.8)	0.790
Up to complete HS	401 (85.3)	256 (63.8)	105 (26.2)	40 (10.0)	
Higher than HS	69 (14.7)	47 (68.1)	16 (21.4)	6 (10.1)	
Marital status	470	303 (64.5)	121 (25.7)	46 (9.8)	0.661
With a partner	294 (62.6)	194 (66.0)	73 (24.8)	27 (9.2)	
Without a partner	176 (37.4)	109 (62.0)	48 (27.2)	19 (10.8)	
Income	470	303 (64.5)	121 (25.7)	46 (9.8)	0.796
No income	36 (7.7)	27 (75.0)	7 (19.4)	2 (5.6)	
Up to 1 MW	181 (38.5)	113 (62.4)	52 (28.7)	16 (8.9)	
From 1 a 3 MW	168 (35.7)	109 (64.9)	41 (24.4)	18 (10.7)	
From 3 to 5 MW	43 (9.1)	30 (69.8)	8 (18.6)	5 (11.6)	
More than 5 MW	18 (3.8)	9 (50)	7 (38.9)	2 (11.1)	
Not informed	24 (5.2)	15 (62.5)	6 (25.0)	3 (12.5)	
Self-declared skin color	470	303 (64.5)	121 (25.7)	46 (9.8)	0.382
White	83 (17.7)	44 (53.0)	31 (37.3)	8 (9.7)	
Black	103 (21.9)	67 (65.0)	25 (24.3)	11 (10.7)	
Brown	269 (57.2)	181 (67.3)	62 (23.0)	26 (9.7)	
Asian descendant	13 (2.8)	9 (69.2)	3 (23.1)	1 (7.7)	
Indigenous	2 (0.4)	2 (100.0)	0 (0)	0 (0)	
Delivery route	470	303 (64.5)	121 (25.7)	46 (9.8)	**0.001**
Vaginal	331 (70.4)	230 (69.5) ^a^	77 (23.3)	24 (7.2) ^a^	
Cesarean	139 (29.6)	73 (52.5) ^b^	44 (31.7)	22 (15.8) ^b^	
GA at delivery	466	302 (64.5)	121 (26.0)	45 (9.6)	**<0.001**
Preterm (<37 weeks)	43 (9.2)	6 (14.0) ^a^	10 (23.2)	27 (62.8) ^a^	
Term (>37 weeks)	423 (90.8)	294 (69.5) ^b^	111 (26.2)	18 (4.3) ^b^	
Number of PN consultations	336	215 (64.0)	87 (25.9)	34 (10.1)	0.655
Less than 6	44 (13.1)	26 (59.1)	12 (27.3)	6 (13.6)	
6 consultations or more	292 (86.9)	189 (64.7)	75 (25.7)	28 (9.6)	
Deliveries	470	303 (64.5)	121 (25.7)	46 (9.8)	0.561
1 child	233 (49.6)	146 (62.7)	61 (26.2)	26 (11.1)	
2 children or more	237 (50.4)	157 (66.2)	60 (25.3)	20 (8.5)	
COVID-19 symptoms during pregnancy	468	301 (64.3)	121 (25.9)	46 (9.8)	0.825
Yes	37 (7.9)	25 (67.6)	8 (21.6)	4 (10.8)	
No	431 (92.1)	276 (64.0)	113 (26.2)	42 (9.8)	
Any DM form during pregnancy	339	223 (64.8)	84 (24.8)	32 (9.4)	0.852
Yes	72 (21.2)	49 (68.0)	16 (22.2)	7 (9.8)	
No	267 (78.8)	174 (65.1)	68 (25.5)	25 (9.4)	
Follow-up with a dietician during pregnancy	470	303 (64.5)	121 (25.7)	46 (9.8)	0.784
Yes	86 (18.3)	55 (64.0)	24 (27.9)	7 (8.1)	
No	384 (81.7)	248 (64.6)	97 (25.3)	39 (10.1)	
Exercising	470	303 (64.5)	121 (25.7)	46 (9.8)	0.030
Yes	178 (37.9)	128 (71.9) ^a^	37 (20.8) ^a^	13 (7.3)	
No	292 (62.1)	175 (59.9) ^b^	84 (28.8) ^b^	33 (11.3)	
Smoking	470	303 (64.5)	121 (25.7)	46 (9.8)	0.568
Yes	35 (7.4)	22 (62.9)	11 (31.4)	2 (5.7)	
No	435 (92.6)	281 (64.6)	110 (25.3)	44 (10.1)	
Alcohol intake	470	303 (64.5)	121 (25.7)	46 (9.8)	0.334
Yes	37 (7.9)	26 (70.3)	6 (16.2)	5 (13.5)	
No	433 (92.1)	277 (64.0)	115 (26.5)	41 (9.5)	

* Statistical test: chi-square test with Bonferroni correction. Frequencies followed by different letters between the categories represent a statistically significant difference between groups. HS: High school; DM: diabetes mellitus; GA: gestational age; PN: prenatal; MW: minimum wage. Minimum wage in Brazil at research time was BRL 1045.00 (one thousand and forty-five Reais). *p* values in bold are statistically significant.

**Table 2:** Features of food consumed by pregnant women and their association with birth weight categories.

Variables	Total Sample (n%)	Adequate Weight (n%)	Insufficient Weight (n%)	Low Weight (n%)	*p*-Value *
Beans, peas, chickpeas or legumes	459	294 (64.0)	120 (26.2)	45 (9.8)	0.987
Insufficient	54 (11.8)	35 (64.8)	14 (25.9)	5 (9.3)	
Regular	405 (88.2)	259 (62.0)	106 (27.2)	40 (10.8)	
Vegetables, and/or legumes	458	293 (64.0)	120 (26.2)	45 (9.8)	0.315
Insufficient	117 (25.5)	78 (66.7)	25 (21.4)	14 (11.9)	
Regular	341 (74.5)	215 (63.0)	95 (27.9)	31 (9.1)	
Fresh fruit (fresh fruit juices were not considered)	459	294 (64.0)	120 (26.2)	45 (9.8)	0.296
Insufficient	206 (44.9)	124 (60.2)	60 (29.1)	22 (10.7)	
Regular	253 (55.1)	170 (67.2)	60 (23.7)	23 (9.1)	
Beef, pork, chicken or fish	456	292 (64.0)	120 (26.3)	44 (9.7)	0.043
Insufficient	105 (23.0)	74 (70.5)	18 (17.1)	13 (12.4)	
Regular	351 (77.0)	218 (62.1)	102 (29.1)	31 (8.8)	
Food prepared outside the home	457	292 (63.9)	120 (26.3)	45 (9.8)	0.631
Inadequate	97 (21.2)	58 (59.8)	28 (28.9)	11 (11.3)	
Less than two days a week	360 (78.8)	234 (65.0)	92 (25.5)	34 (9.5)	
Sausages (burger, ham, salami, sausage).	459	294 (64.0)	120 (26.2)	45 (9.8)	
Inadequate	158 (34.4)	105 (66.5)	40 (25.3)	13 (8.2)	0.641
Less than two days a week	301 (65.6)	189 (62.8)	80 (26.6)	32 (10.6)	
Sweetened beverages (sweetened fruit juice, soda, caned juice, powdered juice)	458	293 (64.0)	120 (26.2)	45 (9.8)	0.428
Inadequate	303 (66.2)	188 (62.0)	85 (28.1)	30 (9.9)	
Less than two days a week	155 (33.8)	105 (67.7)	35 (22.6)	15 (9.7)	
Instant noodles packaged snacks or salty crackers	457	292 (63.9)	120 (26.3)	45 (9.8)	0.932
Inadequate	160 (35.0)	105 (65.0)	41 (25.5)	15 (9.4)	
Less than two days a week	297 (65.0)	188 (63.3)	79 (26.6)	30 (10.1)	
Stuffed biscuits and sweets (candy, chewing gum, gelatin, brigadeiro, sweet pies)	456	292 (64.0)	119 (26.1)	45 (9.9)	0.836
Inadequate	184 (40.4)	117 (63.6)	47 (25.5)	20 (10.1)	
Less than two days a week	272 (59.6)	175 (64.3)	72 (26.5)	25 (9.2)	

* Statistical test: chi-square test.

**Table 3:** Multinomial logistic regression model based on odds ratio (OR) estimates and 95%CI associated with birth weight.

Variables	Categories	Insufficient Birth Weight ^a^		Low Birth Weight ^a^	
		OR	Adjusted OR *	OR	Adjusted OR *
Delivery route ^b^	Cesarean section	1.49 (0.86–2.58)	1.47 (0.71–3.06)	2.83 (1.19–6.73)	**7.70** **(2.33–25.4)**
Total weight gain during pregnancy (kg) ^c^		0.94 (0.90–0.98)	**0.94** **(0.90–0.99)**	0.94 (0.88–1.01)	0.94 (0.87–1.03)
Exercising ^d^	No	1.96 (1.15–3.33)	**2.85** **(1.38–5.89)**	1.85 (0.70–4.89)	1.07 (0.31–3.68)
Gestational age at birth ^e^	Preterm	5.03 (1.61–15.6)	**5.59** **(1.53–20.4)**	56.1 (17.2–183.5)	**70.9** **(16.4–305.8)**

^a^ Adequate weight as a reference; ^b^ Vaginal birth as reference; ^c^ Quantitatively assessed weight gain; ^d^ Exercising as reference; ^e^ Birth term as reference. * Adjusted for maternal age, schooling, smoking, alcohol consumption, and diabetes mellitus during pregnancy. Values in bold are statistically significant.

## Data Availability

Restrictions apply to the availability of these data. Data were obtained from a cohort entitled “Childbirth and breastfeeding in children of mothers infected with SARS-CoV-2”, and some of this work is ongoing.
